# Genetic engineering of *Escherichia coli* to improve L-phenylalanine production

**DOI:** 10.1186/s12896-018-0418-1

**Published:** 2018-01-30

**Authors:** Yongfei Liu, Yiran Xu, Dongqin Ding, Jianping Wen, Beiwei Zhu, Dawei Zhang

**Affiliations:** 1 Tianjin Institutes of Industrial Biotechnology, Chinese Academy of Sciences, Tianjin, 300308 People’s Republic of China; 2 Department of Biological Engineering, School of Chemical Engineering and Technology, Tianjin University, Tianjin, 300072 People’s Republic of China; 3 Key Laboratory of Systems Microbial Biotechnology, Chinese Academy of Sciences, Tianjin, 300308 People’s Republic of China; 4 School of Food Science and Technology, National Engineering Research Center of Seafood, Dalian Polytechnic University, Dalian, 116034 People’s Republic of China

**Keywords:** L-phenylalanine, Metabolic engineering, Proteomics, TyrR, AroD

## Abstract

**Background:**

L-phenylalanine (L-Phe) is an essential amino acid for mammals and applications expand into human health and nutritional products. In this study, a system level engineering was conducted to enhance L-Phe biosynthesis in *Escherichia coli*.

**Results:**

We inactivated the PTS system and recruited glucose uptake via combinatorial modulation of *galP* and *glk* to increase PEP supply in the Xllp01 strain. In addition, the HTH domain of the transcription factor TyrR was engineered to decrease the repression on the transcriptional levels of L-Phe pathway enzymes. Finally, proteomics analysis demonstrated the third step of the SHIK pathway (catalyzed via AroD) as the rate-limiting step for L-Phe production. After optimization of the *aroD* promoter strength, the titer of L-Phe increased by 13.3%. Analysis of the transcriptional level of genes involved in the central metabolic pathways and L-Phe biosynthesis via RT-PCR showed that the recombinant L-Phe producer exhibited a great capability in the glucose utilization and precursor (PEP and E4P) generation. Via systems level engineering, the L-Phe titer of Xllp21 strain reached 72.9 g/L in a 5 L fermenter under the non-optimized fermentation conditions, which was 1.62-times that of the original strain Xllp01.

**Conclusion:**

The metabolic engineering strategy reported here can be broadly employed for developing genetically defined organisms for the efficient production of other aromatic amino acids and derived compounds.

**Electronic supplementary material:**

The online version of this article (10.1186/s12896-018-0418-1) contains supplementary material, which is available to authorized users.

## Background

L-Phenylalanine is an essential and commercially available aromatic amino acid that has been widely used in pharmaceuticals and as food additive [1, 2]. More than 30,000 tons of L-Phe are needed annually to meet the increasing demand for the low-calorie sweetener aspartame [3]. Moreover, L-Phe is extensively used in pharmaceutically active compounds, such as anti-inflammatory drugs [4], central nervous system neuropeptides, and HIV protease inhibitors [2]. Due to the rapid development of metabolic engineering, it is possible to rationally design the genetic circuits thus producing L-Phe in microorganisms [5, 6]. In fast-growing bacteria such as *Escherichia coli* or *Corynebacterium glutamicum*, high titers of L-Phe (57 g/L and 50 g/L, respectively) can be achieved via metabolic network optimization [7, 8].

In *E. coli*, the L-Phe synthetic pathway has two precursors: phosphoenolpyruvate (PEP) and erythrose-4-phosphate (E4P), which were generated from the Embden-Meyerhof-Parnas (EMP) pathway and pentose phosphate (PP) pathway, respectively. However, more than 50% of PEP was used for the translocation and phosphorylation of glucose by the phosphotransferase system (PTS) [3]. Replacement of this glucose transport and phosphorylation capabilities of the PTS would be an efficient approach for high PEP availability. Chandran et al. [9] used glucose facilitator and glucokinase (*glf* and *glk*) from *Zymomonas mobilis* for glucose uptake in *E. coli*, thus greatly improving the production of shikimic acid. Meza et al. overexpressed the D-galactose transporter GalP in PTS inactivated *E. coli* and increased the yield of aromatics 10-times [10].

The condensations of PEP and E4P were catalyzed via 3-deoxy-D-arabinoheptulosonate-7-phosphate (DAHP) synthase [10]. This synthase contains three isoforms: AroG, AroH, and AroF, which are subject to transcriptional and allosteric feedback-regulation by L-Phe, L-tryptophan (L-Trp), and L-tyrosine (L-Tyr), respectively. Another bottleneck in the L-Phe synthetic pathway appears in the formation of phenylpyruvate, catalyzed by the bifunctional enzyme chorismate mutase/prephenate dehydratase (PheA). PheA plays a significant role in phynylpyruvate production and this enzyme is feedback inhibited via allosteric binding of L-Phe. To improve the production of L-Phe in *E. coli*, much work has been devoted to deregulating the feedback inhibition of DAHP synthase and PheA to channel the carbon influx from the central metabolism towards L-Phe [3]. Ray et al. [11] found AroH to be insensitive to Trp by replacing Val^147^ to Gly^149^. Tyr-insensitive AroF mutants were obtained by replacing Pro^148^ or Gln^152^ or deletion of Ile^11^. Zhou et al. [12] selected a L-Phe producer with PheA_mut_(T326P) from the N-methyl-N′-nitro-N-nitrosoguanidine (NTG) mutation library and reported that PheA (T326P) exerted a maximum L-Phe titer of 57.63 g/L with high L-Phe productivity (1.5 g/L/h), which was the highest level reported so far. Zhou et al. [8] overexpressed *pheA*^*fbr*^ as well as wild-type *aroF*^*wt*^ in the *E. coli* strain WSH-Z06 and the L-Phe titer of the recombinant strain reached 35.38 g/L in a 3 L fermenter, which was 2.81-times higher than the original strain and the L-Phe yield on glucose (0.26 mol/mol) was two-times higher than that of the initial strain.

The TyrR regulator of *E. coli* is a class I transcription factor. This regulator constitutes more than nine transcription units, each of which is regulated in a distinctive manner by TyrR [13]. TyrR has three functional domains: the N-terminal domain, the central domain, and the C-terminal domain [14]. The N-terminal domain is able to bind aromatic amino acid ligands and interact with the α-subunit of RNA polymerase to activate the transcription of specific genes. The central domain contains an ATP binding domain, which is able to strengthen the affinity between the TyrR protein and the DNA recognition site in the C-terminal domain by nearly 4-times. The C-terminal domain contains α helix-turn-helix (HTH) motif and is responsible for binding to the TyrR box sequence of controlled genes.

The TyrR regulon consists of more than nine transcription units and the regulation mechanisms vary between each individual unit [14, 15]. Up to now, 13 TyrR regulons (including TyrR itself) have been identified by using the method of genomic SELEX (9). These regluons include: AroF, AroL TyrP, Mtr, TyrB, AroP, AroG CusC, CyaA, ProP, FolA, and HolE [15]. These mechanisms were discovered by observing changes of the transcription level of particular genes in either presence or absence of aromatic amino acids and were further validated by observing the results of activation or repression after introducing site mutations into the TyrR [16]. Mutations in the ATP-binding domain affect the Phe-mediated and Tyr-mediated repression of the *aroG* gene [16].

In light of the discussion above, system level engineering was conducted in the L-Phe producer Xllp01. Firstly, we inactivated the PTS system and then co-overexpressed *galP* and *glk* genes to decrease consumption of PEP. In addition, due to the lack of relevant research on the engineering transcription factor TyrR in the industrial *E. coli* strains, we attempted to engineer the HTH domain of the TyrR to investigate the effects of the TyrR mutants on the transcription level of genes involved in the L-Phe biosynthetic pathway. Furthermore, proteomics technology was applied to provide a global protein expression profile of strains by comparing the quantities of proteins between the *E. coli* wild type and the engineered L-Phe producer.

## Methods

### Bacterial strains and cultivation conditions

All strains and plasmids used in this study are listed in Table 1. The xllp01 strain was renamed in this experiment and the genome background of the strain was the same as the HD-1 strain in the publication [17].

**Table 1:** Strains and plasmids used in this study

Strains and plasmid	Genotype	Source or reference
*E. coli* W3110	Wild type *W3110* derived mutant L-tyrosineauxotrophic	Our lab
Xllp01	p15A::pheA-Thr326Pro p15A::aroF carried by PL promoter kan^r^	Our lab
Xllp02	Xllp01 Δ*ptsH*	This work
Xllp03	Xllp02 Pm37-*galp* Pm37-*glk*	This work
Xllp04	Xllp02 Pm37-*galp* Pm93-*glk*	This work
Xllp05	Xllp02 Pm93-*galp* Pm37-*glk*	This work
Xllp06	Xllp02 Pm93-*galp* Pm93-*glk*	This work
Xllp07	Xllp04 *tyrR* S493 T	This work
Xllp08	Xllp04 *tyrR* T495I	This work
Xllp09	Xllp04 *tyrR* N499D	This work
Xllp10	Xllp04 *tyrR* A498V	This work
Xllp11	Xllp04 *tyrR* S482 N	This work
Xllp12	Xllp08 pBR322:: *tktA* carried by trc promoter Amp^r^	This work
Xllp13	Xllp08 pBR322:: *ppsA* carried by trc promoter Amp^r^	This work
Xllp14	Xllp08 pBR322:: *pckA* carried by trc promoter Amp^r^	This work
Xllp15	Xllp08 pBR322:: *talB* carried by trc promoter Amp^r^	This work
Xllp16	Xllp08 pBR322:: *aroD* carried by trc promoter Amp^r^	This work
Xllp17	Xllp08 pBR322:: *ydiB* carried by trc promoter Amp^r^	This work
Xllp18	Xllp08 pBR322:: *aroC* carried by trc promoter Amp^r^	This work
Xllp19	Xllp08 pBR322:: *aroD* carried by BBa_j23109 promoter Amp^r^	This work
Xllp20	Xllp08 pBR322:: *aroD* carried by BBa_j23105 promoter Amp^r^	This work
Xllp21	Xllp08 pBR322:: *aroD* carried by BBa_j23106 promoter Amp^r^	This work
Xllp22	Xllp08 pBR322:: *aroD* carried by BBa_j23101 promoter Amp^r^	This work
Xllp23	Xllp08 pBR322:: *aroD* carried by BBa_j23100 promoter Amp^r^	This work
p-*tktA*	pBR322::*tktA* trc promoter Amp^r^	This work
p-*ppsA*	pBR322::*ppsaA* trc promoter Amp^r^	This work
p-*talB*	pBR322::*talB* trc promoter Amp^r^	This work
p-*pckA*	pBR322::*pckA* trc promoter Amp^r^	This work
p-*aroD*	pBR322::*aroD* trc promoter Amp^r^	This work
p-*ydiB*	pBR322::*ydiB* trc promoter Amp^r^	This work
p-*aroC*	pBR322::*aroC* trc promoter Amp^r^	This work
p-j23100-*arod*	pBR322::*aroD* BBa_j23100 promoter Amp^r^	This work
p-j23101-*arod*	pBR322::*aroD* BBa_j23101 promoter Amp^r^	This work
p-j23106-*arod*	pBR322::*aroD* BBa_j23106 promoter Amp^r^	This work
p-j23105-*arod*	pBR322::*aroD* BBa_j23105 promoter Amp^r^	This work
p-j23109-*arod*	pBR322::*aroD* BBa_j23109 promoter Amp^r^	This work
pKD46	applied for gene knocking-out	[18]
pDS132	applied for gene knocking-out	[19]
P15A	applied for gene overexpression	[26]

The culture media used in this study included Luria-Bertani (LB) liquid culture medium (10 g/L NaCl, 5 g/L yeast extract, and 10 g/L Tryptone), M9 media broth (1 g/L NH_4_Cl, 6.5 g/L Na_2_HPO_4_, 3.5 g/L KH_2_PO_4_, 40 g/L glucose, 0.24 g/L MgSO_4_, and 0.01 g/L CaCl_2_), and recovery broth (20 g/L Tryptone, 5 g/L Yeast Extract, 0.5 g/L NaCl, 0.19 g/L KCl, 0.95 g/L MgCl_2_, and 3.6 g/L glucose).

For shake flask fermentation, a single colony was inoculated into 5 mL of LB medium with appropriate antibiotics and cultured overnight at 37 °C. The strains were then inoculated into a 500 mL shake flask containing 20 mL of seed culture medium (8 g/L yeast extract, 14 g/L (NH_4_)_2_SO_4_, 2 g/L sodium citrate, 4 g/L KH_2_PO_4_, 20 g/L glucose, 8 mg/L FeSO_4_•7H_2_O, 40 mg/L thiamine, and 2 g/L MgSO_4_) at a ratio of 1:100 and incubated at 37 °C, 220 rpm for 8 h. After this, the seed culture was transferred into the fermentation medium (20 g/L glucose, 10 g/L (NH_4_)_2_SO_4_, 5 g/L KH_2_PO_4_, 5 g/L MgSO_4_, 4 g/L yeast extract, 0.015 g/L FeSO_4_•7H_2_O, 0.015 g/L MnSO_4_•H_2_O, 3 g/L betaine, 10 μg/L biotin, and 250 mg/L Tyr) at a ratio of 1:30. The corresponding antibiotics were added into the culture medium. The cultivation was performed in triplicate for each strain.

To cultivate strains in a 5 L fermenter, strains that were cultivated in LB overnight were inoculated into the seed culture for 7 h at 37 °C and then inoculated into a 5 L fermenter containing 3.5 L seed culture medium (1:10, *v*/v). The pH was controlled at 7.0 ± 0.1 via automatic addition of a 25% ammonia water, and the dissolved oxygen (DO) was constantly retained at 40% saturation by cascading the agitation speed (400–1000 rpm) and aeration rate (2–5 vvm). Glucose feeding was performed with a peristaltic pump when the initial glucose in the medium was exhausted and the concentration of glucose maintained below 5 g/L. Samples were collected every 2 h to determine cell density (OD_600_), residual glucose, off-line pH, amino acid concentrations (after 16 h), and plasmid stability.

### Amplification and overexpression genes

For gene overexpression, target genes were amplified from the genomic DNA of Xllp01 and inserted into the pBR322 plasmid. PCR was employed to introduce endonuclease restriction sites at the end of the gene fragments by using the primers shown in Additional file 1: Table S1. Genomic editing described by Datsenko and Wanner [18] was used to obtain *E. coli* derived strains in this research. The sacB-flanked chloramphenicol-resistance gene was amplified from plasmid pDS132 and the upstream and downstream homology of target genes were amplified from W3110 genome. Then the three fragments were then fused together by fusion PCR [19]. Site mutations on TyrR were achieved by using the technique of rapid PCR site-directed mutagenesis in vitro (KOD-Plus-Mutagenesis Kit, TOYOBO). Fragments were transformed into *E. coli* via electrotransformation.

### Analysis of L-Phe production using HPLC

L-Phe titers were measured using high-performance liquid chromatography (HPLC) with UV detection. Samples collected from either shake flasks or 5 L fermenter were centrifuged and the supernatants were diluted with the required amount of ddH_2_O. The L-Phe concentration was determined via HPLC according to the instruction of Zorbax Eclipse-AAA columns on an Agilent 1100 HPLC. This column could achieve the resolution ratio at approximately 10 pmols. Derivatization was performed according to the manufacturer’s protocol. L-Phe was separated by using mobile phase A (40 mM Na_2_HPO_4_ pH 7.8) and phase B (Acetonitrile (ACN): MeOH: H_2_O = 45:45:10) with gradient elution (The gradient variation ratio of phases A:B with time was shown in Additional file 1: Table S2). Column temperature was set to 30 °C and the flow rate was 2 mL/min. One sample was quantitatively analyzed within 30 min. All experiments were conducted in triplicate.

### Proteomic preparation and digestion

Protein preparation and digestion were performed as follows: 5 mL of fermentation broth (at logarithmic early growth) was centrifuged for 20 min at 3500 g and 4 °C. The supernatants were washed with PBS buffer thrice and immediately frozen with liquid nitrogen. Cell pellets were then resuspended in a UA buffer (8 M Urea dissolved in 0.1 M Tris–HCl (pH 8.5), containing 1% β-mercaptoethanol (*v*/v), and 10 mM DTT) and broken via sonication cracking on ice (5 s on, 5 s off for a total of 10 min). The samples were centrifuged for 10 min at 2275 g and 4 °C and the resulting supernatant was filtered through a sterile membrane filter (Millipore, PES polymer cast, 0.22 μm). A protein pellet was quantitated using a 2D–Quant Kit (GE Healthcare) and dissolved in digestion buffer (100 mM TEAB (triethylammonium bicarbonate)) to a final concentration of 1 mg/mL. Equal aliquots were digested with trypsin (Promega) overnight at 37 °C and then lyophilized [20, 21].

### Proteomic data analysis

Mass spectrum analysis was conducted via a Triple Time of Flight (TOF) 5600 mass spectrometer (AB SCIEX, USA). A NanoLC pre-column (Chromxp C18-LC-3 μm, Eksigent) and an analytical column (C18-CL-120, Eksigent) were separately used to trap and elute peptides via gradient wash from 5 to 35% Buffer B (Buffer A: 2% ACN, 98% H_2_O, Buffer B: 98% ACN, 2% H_2_O, 0.1% formic acid) at a flow rate of 300 nL/min. Full-scan MS was performed in the positive ion mode with a nano-ion spray voltage of 2.5 kV from 350 to 1500 (m/z), with up to 30 precursors selected for MS/MS if the precursors exceeded a threshold of 125 counts per second. Charged peptides ranging from + 2 to + 5 were screened for the MS/MS analysis. The collision energy (CE) for the collision-induced dissociation (CID) was automatically controlled using an Information-Dependent Acquisition (IDA) CE parameter script to achieve optimum fragmentation efficiency.

To identify the proteins within samples, theoretical peptide spectra of proteins deposited in a protein database were matched to the acquired tandem mass spectra using the search engine MASCOT. Then, the approach was termed spectral counting, implying counting and comparison of the number of fragment-ion spectra (MS/MS) acquired for peptides of a special protein.

### Real-time PCR analysis

Total RNA extraction was conducted via RNA prep pure Cell/Bacteria Kit (DP430, Tiangen, China) and the purified RNA was stored at − 80 °C. cDNA synthesis was performed via PrimeScript® 1st Strand cDNA Synthesis Kit (D6110A, TAKARA, Japan) and the purified cDNA was stored at − 20 °C. Real-time PCR (RT–PCR) was performed with the ABI Prism7000 Sequence Detection System using the SYBR Green PCR Master Mix. The amplification conditions were as follows: 10 min at 95 °C, and a two-step cycle at 95 °C for 15 s and 60 °C for 60 s for a total of 45 cycles (primers used are listed in Additional file 1: Table S3). All samples were performed in triplicate. A non-template control reaction mixture was included for each gene as negative control. All data were normalized using the *ihfB* gene as an internal control (housekeeping gene) [22].

## Results

### Improving the available intracellular PEP by inactivating the PTS system

Previous research has shown that the glucose transportation and phosphorylation capabilities of the PTS system could be replaced by co-overexpressing alternative enzymes D-galactose transporter (GalP) and glucokinase (Glk) to allow higher PEP availability for L-Phe formation [23]. In this case, we attempted to reproduce this approach and determine the appropriate expression levels of both enzymes.

**Fig. 1:**
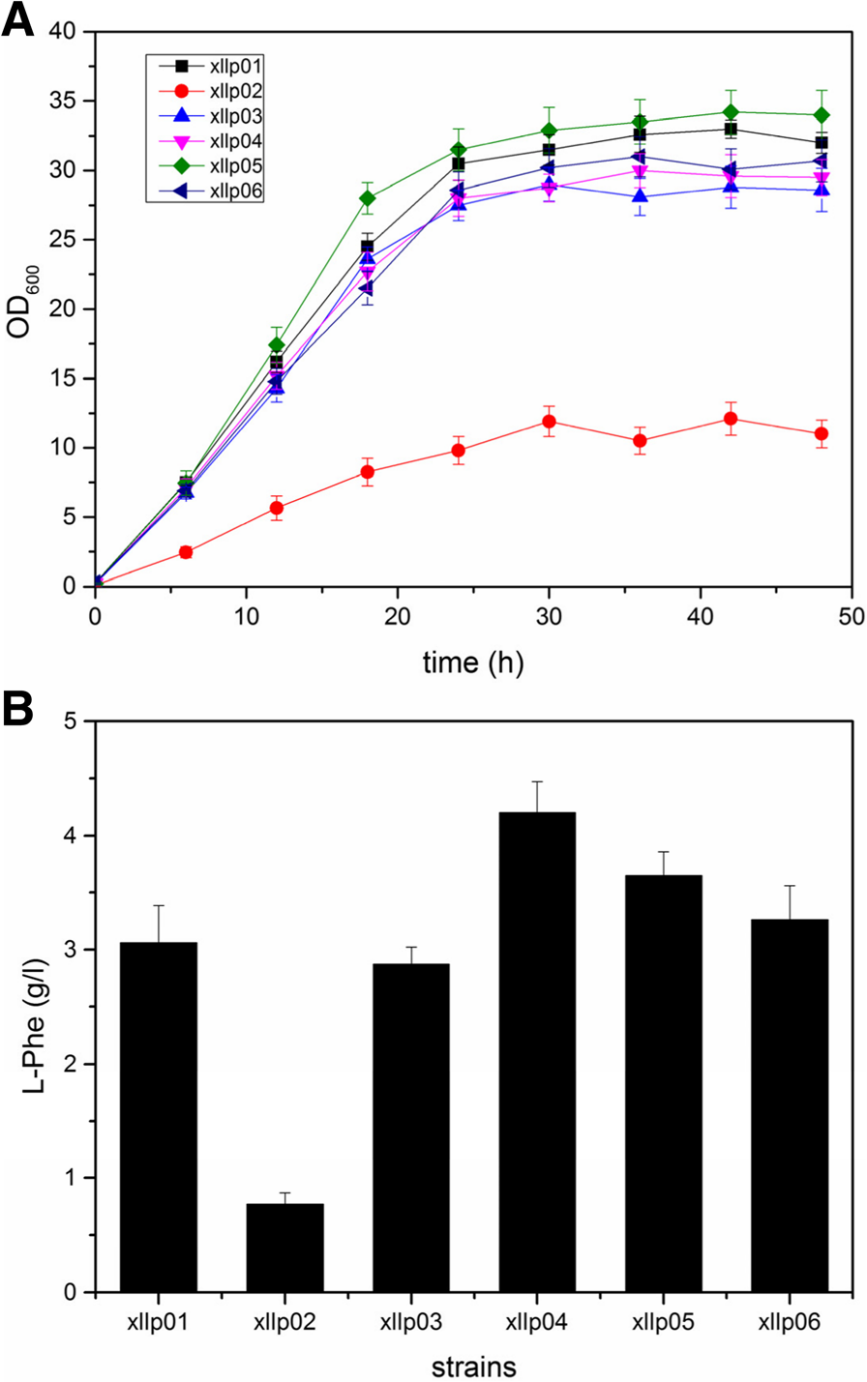
Growth curves and fermentation results of the recombinant strains combinational modulating the *galp* and *glk* genes with different promoters. **a** Growth curves of the recombinant strains; **b** The fermentation results of the recombinant strains in shake flasks. The experiment was repeated three times, and measurements are represented as means with their standard deviation

The Xllp02 strain was created by deleting the *ptsH* gene in Xllp01 strain. As expected, the cells growth of Xllp02 was dramatically decreased in shake flask fermentation due to the lack of sufficient glucose intake (Fig. 1a). A previous study [23] applied several artificial promoters exhibiting different strength for modulating the expression of *galp* and *glk*. Similarly, two promoters Pm93 and Pm37 were selected to combinatorially modulate the expression of both genes thus maintaining the proper specific growth rate and glucose utilization of the recombinant strains. To enhance glucose uptake in Xllp02, two artificial promoters exhibiting different strength Pm37 and Pm93 [23] were selected to combinatorially modulate *galP* and *glk* gene expression in chromosome. Four engineered strains, in which *galP* and *glk* were separately controlled by Pm37 and Pm93 (Table 1), were constructed to test the growth curve, L-Phe titer, and yield. Fig. 1a showed that the growth rate of four strains recovered at different degrees compared to the Xllp02 strain. Interestingly, a good positive correlation was observed between L-Phe production and the growth of strains. This may be due to the unanimous demand of the carbon resourc e for L-Phe production and cell growth. The highest titer of L-Phe was observed in the Xllp04 strain (4.2 g/L), in which *galP* and *glK* were separately modulated by m37 and m93 (Fig. 1b).

### Engineering the HTH of TyrR to optimize L-Phe synthetic pathway

Although the TyrR regulator has evolved a diverse range of specific regulations on the genes involved in L-Phe synthesis, it is difficult to relieve such inhibition by knocking out *tyrR.* Our results showed that the L-Phe production decreased by 32% when *tyrR* gene was knocked out in the Xllp04 strain (Additional file 1: Figure S1). To solve this problem, we attempted to engineer the HTH structure in the C-domain of the TyrR protein to relieve the TyrR’s repression, as several enzymes were up-regulated in the *tyrR* mutation strains [13]. The mutation site was verified via PCR and sequence analysis (Additional file 1: Figure S2).

**Fig. 2:**
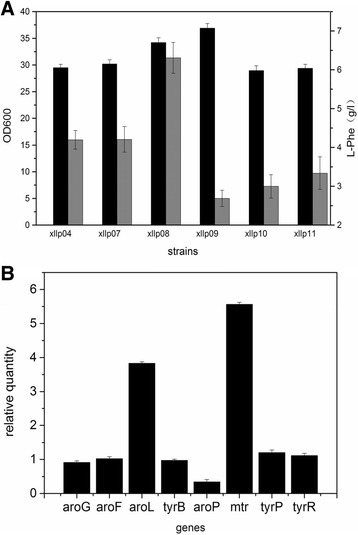
Fermentation results and transcriptional analysis of the TyrR mutant strains. **a** Fermentation results of the TyrR mutant strains in shake flasks. Black columns stands for OD_600_ and gray columns stands for the L-Phe production. **b** Real-time PCR analysis consequences of the genes regulated by the TyrR protein in Xllp08 contrast to Xllp04. Experiments were conducted in triplicate

The replaced residues were located in the second α-helix of the HTH motif, which might be expected to specifically interact with key bases in the TyrR boxes in the promoter region of the regulated genes. The amino acids at position 1, 2, and 6 had been suggested to be involved in specific contacts with the promoters. Therefore, we attempted to change these amino acids to observe activation or repression effects on the genes involved in the L-Phe synthetic pathway. Five potential site mutations were separately introduced into the HTH domain of the TyrR protein in the chromosome of Xllp04 strain, generating Xllp07 (S493 T), Xllp08 (T495I), Xllp09 (N499D), Xllp10 (A498V), and Xllp11 (S482 N), respectively. After fermentation, the five strains presented a similar trend of growth, but showed significant differences on the titer of L-Phe (Fig. 2a). The Xllp08 strain showed an apparent increase in L-Phe production and the titer reached 6.31 g/L, which was approximately 1.5-times of that of Xllp04. To investigate the hyperproducing mechanism of this strain, a quantitative PCR (qPCR) analysis was conducted to monitor differences in transcriptional level of genes regulated by TyrR protein between Xllp08 and Xllp04.

As shown in Fig. 2b, *aroL* (coding shikimate kinase), which was directly involved in the L-Phe metabolism was up-regulated 3.83-times in Xllp08. In addition, the expression of the *mtr* increased 5.56-times. This could contribute to the enhancement of tryptophan uptake, thus reducing the requirement of tryptophan during the fermentation process [24]. The aromatic amino acid transporter *aroP* decreased 0.71-time in Xllp08. Other genes (*tyrB*, *aroG*, *tyrR*, and *tyrP*) did not significantly change in the transcriptional level between both strains.

### Enhancing the L-Phe synthetic pathway flux via proteomics analysis

**Fig. 3:**
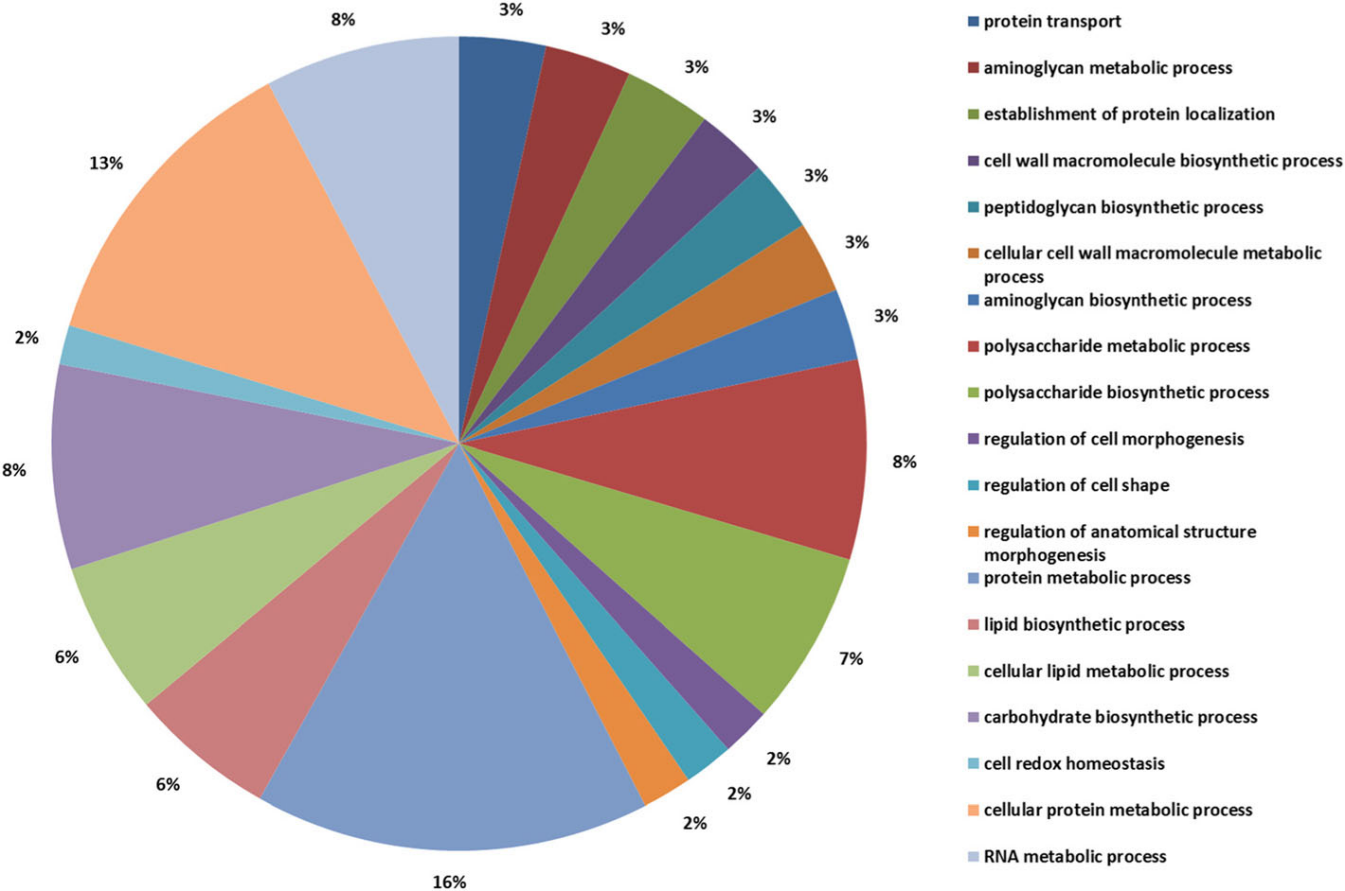
Gene ontology analysis of the proteins identified by the proteomics

**Fig. 4:**
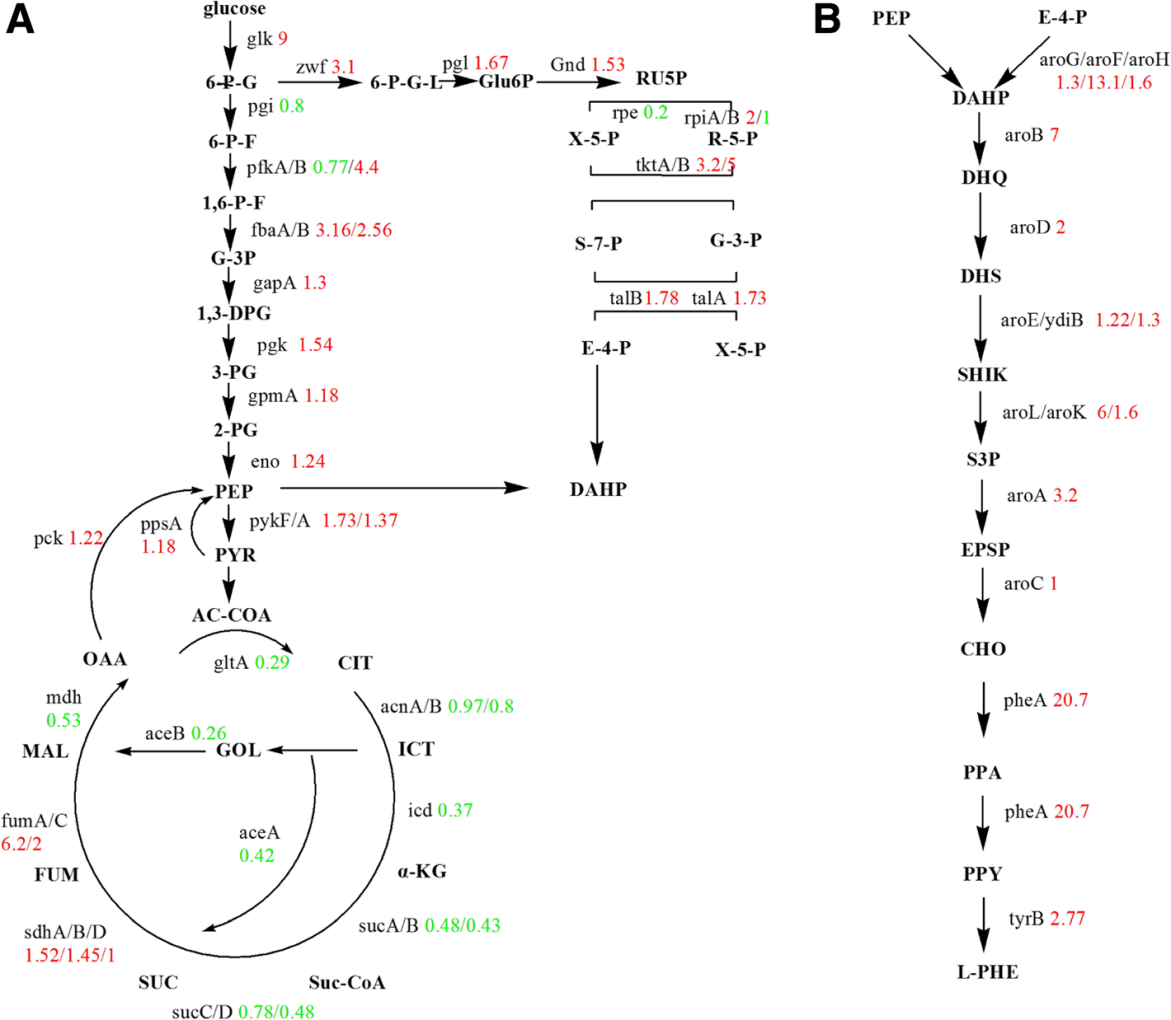
Proteomic analysis of the genes in Xllp08 and wild-type *E. coli* W3110*.* **a** Comparison of central carbon metabolism between wild-type W3110 and Xllp08. Two precursors (PEP and E4P) of the L-Phe synthesis were located in the EMP pathway and PP pathway respectively, the distribution of which would directly affect the L-Phe production and yield. The red data meant upregulation and green data meant downregulation. **b** The great disparity between two studied strains in the aromatic amino acid synthesis pathway. This figure showed comparion of enzymes in the L-Phe generating pathway between Xllp08 strain and wild-type *E. coli* W3110

To determine the targets for further engineering of the Xllp08 strain, label-free proteomics was performed to investigate differences in protein expression profiles between the Xllp08 and the *E. coli* W3110 strain. Protein samples were collected during the early logarithmic growth and subjected to proteomic analysis. The strict confidence criteria for identification of a total of 1447 proteins were identified in this experiment and roughly represented 40% of the total predicted proteins in *E. coli*. W3110 (Fig. 3). Among the identified proteins, 720 proteins presented increased expression level, while the expression level of 705 proteins was decreased in the Xllp08 strain. In terms of the number of proteins that were identified in each functional category, the most frequently detected category was “Cellular protein metabolic process”, supplying roughly 16% of the entire proteins identified in the proteomics analysis. By setting the criteria for a cutoff of *p* value below 0.05, we focused on the proteins involved in the synthesis of glucose to L-Phe. As shown in Fig. 4a, enzymes in the EMP and PP pathways were generally up-regulated, while enzymes in the TCA cycle were down-regulated in the Xllp08 strain. This indicated that sufficient supply of precursors (PEP and E4P) is of vital importance for increased L-Phe production. In contrast, the TCA cycle seemed virtually irrelevant to the biosynthesis of L-Phe in the Xllp08 strain, which agreed with a previous study [25]. We assumed that the lower expression level in the TCA cycle ensured that sufficient PEP could be channeled into L-Phe production. Therefore, we attempted to overexpress genes encoding PEP and E4P synthesis enzymes (*tktA, talB, pckA*, and *ppsA*) in Xllp08 to increase the accumulation of precursors of L-Phe. However, all of these recombinant strains showed a severe growth reduction without obvious increase in L-Phe production (Additional file 1: Figure S3). We assumed that carbon flux between the PP pathway and TCA cycle reached a relative balance for cell growth and L-Phe production in Xllp08, and overexpressing either of them would cause a severe disturbance in cells, which acted as a metabolic overload for L-Phe production.

**Fig. 5:**
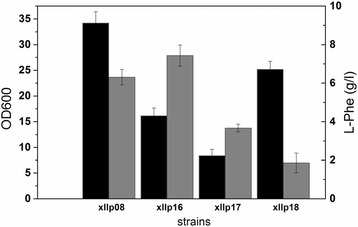
Fermentation results of the Xllp08 strains and its derivative strains. Xllp16, Xllp17, and Xllp18 were obtained by overexpressing *aroD*, *ydiB* and *aroC* in Xllp08 strains, respcetively. The black column meant OD_600_ and the gray column meant L-Phe titer. Experiments were conducted in triplicate

**Fig. 6:**
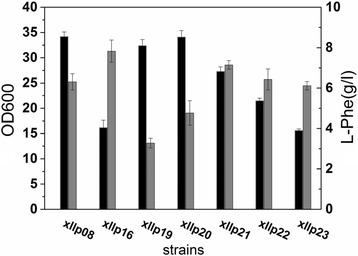
Fermentation results of the recombinant strains overexpressing *aroD* gene with different promoters in Xllp08 strain. The black column meant OD_600_ and the gray column meant the L-Phe titer. The expreiments were repeated three times

In addition to AroF and PheA which were overexpressed in plasmid p15A [26], other enzymes in the SHIK pathway such as AroB, AroK, AroL, AroA, and TyrB were also significantly increased in Xllp08 (Fig. 4b). Surprisingly, AroD (2.0-times), YdiB (1.3-times), and AroC (1.02-times) were not up-regulated as dramatically as other enzymes, suggesting that these steps may be bottlenecks in Xllp08. To confirm this, *aroD*, *ydiB*, and *aroC* were separately ligated in the pBR322, and transformed into the Xllp08 strain thus producing Xllp16, Xllp17, and Xllp18. The fermentation results of these strains are shown in Fig. 5. The L-Phe titers of Xllp17 (3.67 g/L) and Xllp18 (1.86 g/L) remained flat or falling compared to that of the Xllp08. In contrast, a prominent increase in L-Phe production was observed in Xllp16 (7.43 g/L); however, the final OD was only 47.3% that of Xllp08. A trade-off exists between the accumulation of biomass and the production of target compounds in industrial microorganisms [27]. Therefore, it was necessary to optimize the expression levels of *aroD* to further increase the L-Phe titer of Xllp08. To solve this problem, five promoters exhibiting different strength were chosen from the MIT parts registry (http://parts.igem.org/) to overexpress *aroD* in the Xllp08 strain. The relative strength of the selected promoters was given in Additional file 1: Figure S4. The final OD of the recombinant strains presented a decreasing trend accompanied with an increase of promoter strength, while the L-Phe production initially increased and remained stable with a further increase of *aroD* expression level (Fig. 6). In light of the biomass and the titer and productivity of L-Phe, the Xllp21 strain was chosen as the optimized strain for further engineering.

### Transcription analysis of the recombinant strain

**Fig. 7:**
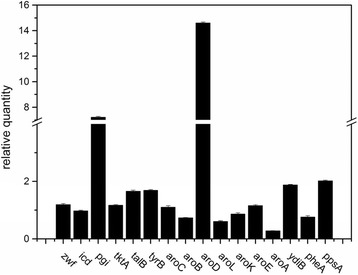
Relative transcription level of genes in Xllp21 and Xllp08 by real-time PCR analysis. Experiments were conducted in triplicate, and measurements are represented as means with their standard deviation

To observe the relevant genic expression disturbance via overexpression of *aroD* in Xllp21, a subset of 16 genes, including genes involved in L-Phe biosynthesis, glucose utilization, and L-Phe precursor metabolism were selected for analysis via RT-PCR [22, 28]. As shown in Fig. 7, the most notable difference appears in the *pgi* gene, with a 7.21-times increase in the Xllp21 strain compared with Xllp08. The *zwf, tktA* and *talB* were also up-regulated 1.19-times, 1.17-times, and 1.66-times, respectively. This indicated that the metabolic flux of glycolysis and pentose phosphate pathway was enhanced. The *icd* gene showed no apparent change (0.97-time) while the *ppsA* gene coding for phosphoenolpyruvate synthase (which stood for gluconeogenesis pathway from malic acid (MAL) to pyruvic acid (PYR) and PEP) showed a 2.02-times increase. This would facilitate more PEP to pour into the L-Phe synthetic pathway and contribute to increasing the conversion ratio from glucose to L-Phe [29]. Therefore, it can be concluded that the Xllp21 strain was engineered to channel more carbon influx from the central metabolism towards the shikimate pathway by enhancing EMP and PP pathway, while converting flux in the TCA cycle into PEP.

Compared to Xllp08, the transcriptional level of genes *tyrB* and *ydiB* were significantly increased in Xllp21. Other genes of the L-Phe synthetic pathway, such as *aroC, aroB, aroK, aroE,* and *pheA* showed no apparent differences between both strains. In contrast, *aroL* and *aroA* genes showed a 0.61-time and a 0.28-time decrease, respectively. We suggest that the downregulation of *aroA* was due to the lack of sufficient intracellular PEP, which is necessary for this reaction catalyzed by AroA. In light of this, further study should be conducted on the fine-tuning of the entire enzymes in the L-Phe synthetic pathway.

### L-Phe production in a 5 L fermenter

**Fig. 8:**
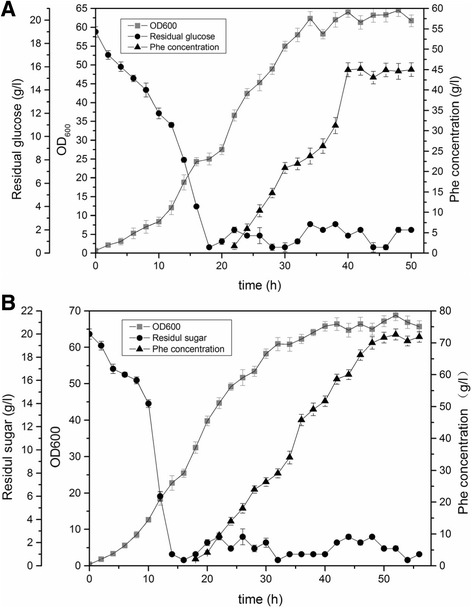
Time course of L-Phe production by strain Xllp01 (**a**) and Xllp21 (**b**) in a 5 L fermenter. Dots meant residual glucose concentration, blocks meant the OD_600_ and triangles meant the L-Phe concentration. The final L-Phe yield and titer was basically constant among the three replicates and one of the three times was given as an example to show the fermentation results

We performed a fed-batch culture with intermittent glucose feeding to investigate the production of L-Phe by the Xllp21 strain (Fig. 8, Additional file 2). Compared to the original strain Xllp01, the final biomass of Xllp21 showed no significant difference. However, the glucose consumption rate of Xllp21 in the lag phase was much faster than that of Xllp01, which indicates to contribute to L-Phe accumulation. For Xllp21, L-Phe started to accumulate in the exponential growth phase after 18 h of cultivation, which is 4 h earlier compared to Xllp01. The final L-Phe production of Xllp21 was 72.9 g/L at 52 h, with a productivity of 0.26 g/g glucose. Plasmid stability was assessed after fermentation by testing the resistance to the ampicillin antibiotic marker. More than 96% of the cells retained ampicillin resistance, indicating that the plasmid p-j23106-arod remained stable throughout cultivation. Compared to the parent strain Xllp01 (Fig. 8a), the L-Phe titer was improved by 62%. Moreover, the yield of glucose was also greatly improved by 39.7% compared to the parent strain (0.186 g/g glucose).

## Discussion

In order to facilitate the comparison with other strains, we summarized all fermentation values (including OD, L-Phe titer and yield on glucose) of all recombinant strains in this study in Table 2.Through the system level engineering, we obtained a high L-Phe producer Xllp21, which was capable of producing 72.6 g/L L-Phe with a yield of 0.26 g/g glucose under the non-optimized fermentation condition. As far as we know, the titer of Xllp21 has exceeded the highest titer of L-Phe reported previously (57.63 g/L [8]) and was comparable to the highest yield (0.27 mol/mol glucose [30]) reported.

**Table 2:** Fermentation values of the recombinat strains

Strains	Titer of Phenylalanine (g/L)	OD_600_	Yield (to glucose g/g)
Xllp01	3.06 (45)	32.5	0.07 (0.18)
Xllp02	0.77	11.6	0.04
Xllp03	2.87	28.7	0.06
Xllp04	4.2	29.6	0.09
Xllp05	3.65	34.1	0.08
Xllp06	3.26	30.3	0.07
Xllp07	4.21	30.2	0.09
Xllp08	6.31	34.2	0.12
Xllp09	2.69	36.9	0.06
Xllp10	2.98	28.9	0.07
Xllp11	3.34	29.4	0.07
Xllp12	1.18	15.9	0.05
Xllp13	3.69	14.1	0.08
Xllp14	1.74	15.3	0.05
Xllp15	1.68	25.9	0.04
Xllp16	7.43	16.2	0.15
Xllp17	3.67	8.4	0.08
Xllp18	1.86	25.2	0.04
Xllp19	3.28	32.4	0.07
Xllp20	4.77	34.1	0.11
Xllp21	7.15 (72.6)	27.3	0.16 (0.26)
Xllp22	6.43	21.5	0.14
Xllp23	6.12	15.6	0.13

Note: All datas were colleted from the shake flask fermentation and calcuated by triplicate samples. Data in the parenthesis was colleted from a 5-L fermenter

In a PTS^−^ strain, the uptake and phosphorylation of glucose could be achieved by combinatorially overexpressing the *galP* and *glk*. A previous study [23] provided several artificial promoters for *galp* and *glk* modulation exhibiting at different strength. By applying this strategy, a high glucose uptake rate was obtained during the fed-batch fermentation of the Xllp04 strain (Fig. 1a). In light of the industrial production process, we avoided to use a weak promoter because the low expression level of *galP* and *glk* would hamper glucose uptake, resulting in a low specific growth rate. In addition, it was found that the expression level of *galP* was predominant for effective glucose uptake in our industrial strain because the final OD_600_ of the strains increased accompanied by an increase of the promoter strength *of galP*. However, higher cell growth did not lead to a higher L-Phe titer. The fermentation result (Fig. 1b) showed that a combinatorially expressing of *galP* and *glk* by using m37 and m93 resulted in the highest L-Phe titer, which was higher than the production of Xllp06 using both strong promoters to control *galP* and *glk*. This indicated that the expression levels of these two genes should be fine-tuned to reach optimal glucose utilization.

In *E. coli,* the synthesis and transport of aromatic amino acids are regulated by the transcription factor TyrR. The C-terminal region of the TyrR protein contains a DNA-binding domain, allowing it to bind specific TyrR box sequences in regulated genes. Previous research showed that the activity of enzymes that are involved in aromatic amino acid biosynthesis altered when mutagenesis was introduced into the C-terminal HTH motif of TyrR [16]. Here, we selected five potential mutants that may deregulate the inhibition effects of *aroG*, *tyrB*, and *aroL*. Interestingly, the five mutant strains exhibited distinct trend of cell growth and L-Phe production, indicating the mechanism of TyrR regulation in *E. coli* to be far more complex than expected. It is likely that the site mutation in the HTH motif might have influenced the interaction with other binding sites associated with unknown genes, which were responsible for cell growth. Transcriptional analysis of Xllp08 showed that this mutation (T495I) caused an increase in the transcriptional level of the *mtr* and *aroL*. It is possible that the mutant on the HTH motif changed the affinity between TyrR regulator and the TyrR box on the promoters of these genes, which led to effective binding of the RNA polymerase to the P_mtr_ and P_aroL_, thus enhancing the transcription level of these genes. Further research is required to reveal the mechanism between TyrR and nucleic acid through which application of this regulator can be improved.

Due to the significant role of PEP, many manipulations of the *ppsA* gene have been conducted to improve the enzymatic properties [22, 23]. However, overexpressing the *ppsA* gene in Xllp08 failed to improve the production of L-Phe due to metabolic overload. Interestingly, the *aroD* overexpressing strain showed an additional *ppsA* up-regulation. We assumed that a great demand for a certain metabolite could in turn accelerate its biosynthesis. Furthermore, another potential strategy for improving available intracellular PEP was to inactivate the PEP-consuming enzymes PykA or PykF. This strategy had been applied to enhance the additional availability of PEP for the synthesis of shikimic acid in *E. coli* [31]. Strains with deleted *pykA* and *pykF* could obtain higher concentrations of intracellular PEP to be channeled into the L-Phe synthetic pathway.

It was necessary to add antibiotics in the feed-batch fermentation. In this study, 100 μg/mL ampicillin was added in the initial of fermentation in a 5 L fermentor. Given that the ampicillin antibiotic doesn’t work for more than 16 h, we replenished the ampicillin antibiotic after 16 h of fermentation to prevent the bacterial contamination and to maintain the plasmid stability. After that it was not necessary to add ampicillin antibiotic into the fermentor because our engineered strain was so competitive that it was almost impossible to result in bacterial contamination.

As to the resistance loss, we assumed that the optical density of cells were so high that 100 μg/mL ampicillin antibiotic couldn’t sufficiently maintain all cells’ plasmid stability (In fact, the final OD of strains could reach to 70~ 75 in a 5 L fermentor). However, overdose of ampicillin will bring damage to the physiology of cells, which will bring about the productivity loss. On the whole, we determined to add 100 μg/mL ampicillin antibiotic into the medium, although 4% of the cells in the 5 L fermenter was found to lose the ampicillin resistance at the end of the fermentation, the final titer and yield of L-Phe was not obviously affected by the plasmid loss of a small number of producers.

## Conclusions

In this study, the glucose utilization system was reconstructed by modulating the *galp* and *glk* genes in a PTS^−^ strain to decrease the PEP consumption. The TyrR regulator was also investigated to modify the transcriptional inhibition in the aromatic amino acid metabolism. The T495I mutation introduced in the DNA binding domain of TyrR greatly altered binding the *aroL* and *mtr* genes, thus intensifying the synthetic pathway and reducing the by-product. Furthermore, the protein expression in the central metabolism and the L-Phe synthetic pathway were observed via proteomic analysis. This study showed that the third step of the SHIK pathway was a bottleneck step for L-Phe production in our recombinant strain. The fine-tuned overexpression of the enzyme AroD improved the glucose uptake rate and gluconeogenesis pathway, thus increasing the production of L-Phe in an already advanced production strain. The detailed characterization presented here provides a systematic approach for designing an engineered strain capable of producing L-Phe, which will be useful for further metabolic engineering.

## Additional files

Additional file 1: Table S1. Primers used in the genetic engineering. **Table S2.** The gradient variation ratio of phases A:B in HPLC analysis of L-Phe concentration. **Table S3.** Primers used in the Real-time PCR analysis. **Figure S1.** Fermentation consequence of the *tyrR* knocked out strain. The gray column means OD_600_ and the black column means L-Phe titer. **Figure S2.** Sequence alignment results of the TyrR_wt_ and TyrR_mut_. This result was obtained by DNAMAN software. **Figure S3.** Overexpression of the precursors’ synthesis encoded genes. The gray column means OD_600_ and the black column means L-Phe titer. **Figure S4.** Relative strength of five promoters that were used to overexpress AroD in xllp08. (DOCX 582 kb)

Additional file 2. Online data of the fermentation of strain Xllp01 and Xllp21. (XLSX 431 kb)
