# Supplementary material for: Genetic engineering of Escherichia coli to improve L-phenylalanine production
Source: BMC Biotechnol. 2018 Jan 30;18:5. doi: 10.1186/s12896-018-0418-1 (PMC5791370; doi:10.1186/s12896-018-0418-1)

**Additional file 1 for**

**Genetic engineering of *Escherichia coli* to improve L-Phenylalanine production**

Yongfei Liu^1#^, Yiran Xu^1,2,3#^, Dongqin Ding^1^, Jianping Wen^2^, Beiwei Zhu^4^, Dawei Zhang^1,3,4 *^

*Corresponding authors. zhang_dw@tib.cas.cn, +86-22-24828749

Email address for all authors:

[liu_yf@tib.cas.cn](mailto:liu_yf@tib.cas.cn); [xu_yr@tib.cas.cn](mailto:xu_yr@tib.cas.cn); [ding_dq@tib.cas.cn](mailto:ding_dq@tib.cas.cn); [jp_wen@126.com](mailto:jp_wen@126.com); zhubeiwei@163.com

1 Tianjin Institutes of Industrial Biotechnology, Chinese Academy of Sciences, Tianjin 300308, P. R. China

2 Department of Biological Engineering, School of Chemical Engineering and Technology, Tianjin University, Tianjin, 300072, P. R. China

3 Key Laboratory of Systems Microbial Biotechnology, Chinese Academy of Sciences, Tianjin 300308, P. R. China

4 School of Food Science and Technology, Dalian Polytechnic University, National Engineering Research Center of Seafood, Dalian, 116034, P. R. China

# These authors contribute equally to this work.

**Supplementary Table 1** Primers used in the genetic engineering

| Genes | Primer name | Primer sequence(5’-3’) |
| --- | --- | --- |
| *ptsH* | ptsH -up-F  ptsH -up-R  ptsH -down-F  ptsH -down-R  ptsH -blank-F  ptsH -blank-R | ttgattcagcctgtcggaactggta  taatagtgaacggcaggtatatgtgtgtatttccccaacttataggttta  cgatgagtggcagggcggggcgtaatttcccgggttcttttaaaaatcag  ccagctcctcatcttcgagcagcat  taaacctataagttggggaaatacatttcccgggttcttttaaaaatcag  ctgatttttaaaagaacccgggaaatgtatttccccaacttataggttta |
| *galP* | galP -m37-F  galP -m93-F  galP -R  galP -up-F  galP -up-R  galP -up-m37-R  galP -up-m93-R  galP -down-F  galP -down-m-F  galP -down-R | ttatctctggcggtgttgacaagagataacaacgttgatataattgagccactggctcgtaatttattgtttaaaccaggaaacagctatgcctgacgctaaaaaacaggggc  ttatctctggcggtgttgacaagagataacaacgttgatataattgagcccgtattgttagcatgtacgtttaaaccaggaaacagctatgcctgacgctaaaaaacaggggc  ttaatcgtgagcgcctatttcgcgc  accgcagattatcatcacaactgaa  taatagtgaacggcaggtatatgtggatgccctccaatatggttattttt  ctcttgtcaacaccgccagagataagatgccctccaatatggttattttt  ctcttgtcaacaccgccagagataagatgccctccaatatggttattttt  cgatgagtggcagggcggggcgtaatctccccaagcttcctcccatcgcg  gcgcgaaataggcgctcacgattaatctccccaagcttcctcccatcgcg  acttcttcaataaaagcttcactgt |
| *glk* | glk –m37-F  glk –m93-F  glk -R  glk -up-F  glk -up-R  glk -up-m37-R  glk -up-m93-R  glk -down-F  glk -down-R | ttatctctggcggtgttgacaagagataacaacgttgatataattgagccactggctcgtaatttattgtttaaaccaggaaacagctatgacaaagtatgcattagtcggtg  ttatctctggcggtgttgacaagagataacaacgttgatataattgagcccgtattgttagcatgtacgtttaaaccaggaaacagctatgacaaagtatgcattagtcggtg  ttacagaatgtgacctaaggtctgg  ttttcatcaccacaatcagaataag  taatagtgaacggcaggtatatgtgtcttcaactgctccgctaaagtcaa  ctcttgtcaacaccgccagagataatcttcaactgctccgctaaagtcaa  ctcttgtcaacaccgccagagataatcttcaactgctccgctaaagtcaa  cgatgagtggcagggcggggcgtaaatccttccttttatatcgggaggta  cttcttccagcgcctgagcggccat |
| *tyrR* | tyrR -F  tyrR -R  tyrR-S493T-R  tyrR-T495I-R  tyrR-N499D-R  tyrR-A498V-R  tyrR-S482N-R  tyrR -up-F  tyrR -up-R  tyrR -down-F  tyrR -down-R  tyrR -blank-F  tyrR -blank-R | atgcgtctggaagtcttttgtgaag  ttactcttcgttcttcttctgactc  ttactcttcgttcttcttctgactcagaccatattcccgcaacttattggcaatcgcggtatgtgtaacg  ttactcttcgttcttcttctgactcagaccatattcccgcaacttattggcaatcgcgatatgtgaaacg  ttactcttcgttcttcttctgactcagaccatattcccgcaacttatcggcaatc  ttactcttcgttcttcttctgactcagaccatattcccgcaacttattgacaatc  ttactcttcgttcttcttctgactcagaccatattcccgcaacttattggcaatcgcggtatgtgaaacgccgagacgttttgccagtttgcgcgtgttg  ctgacggcacgactcgggattaaag  taatagtgaacggcaggtatatgtggggaaccttcacctgaaaaaagaac  cgatgagtggcagggcggggcgtaagcgcgaatatgcctgatggtgcaac  ctgccgatctgccgttcgcccagtc  gttcttttttcaggtgaaggttcccgcgcgaatatgcctgatggtgcaac  gttgcaccatcaggcatattcgcgcgggaaccttcacctgaaaaaagaac |

**Supplementary Table 2** The gradient variation ratio of phases A:B in HPLC analysis of L-Phe concentration

| **Time (min)** | **%B** |
| --- | --- |
| **0** | **0** |
| **1.9** | **0** |
| **18.1** | **57** |
| **18.6** | **100** |
| **22.3** | **100** |
| **23.2** | **0** |
| **26** | **0** |

**Supplementary Table 3** Primers used in the Real-time PCR analysis

| Gene name | Primer name | Primer sequence(5’-3’) |
| --- | --- | --- |
| *zwf* | zwf-F  zwf-R | ttgcatgattgcgatgtctc  tacgcaggtagaatggcaca |
| *icd* | icd-F  icd-R | gttactatcagggcactccaag  cttcttccgaacacggctta |
| *pgi* | pgi-F  pgi-R | cgaagtggttgagcaggaat  tcacgcagcaggatggagtt |
| *tktA* | tktA-F  tktA-R | ctcggcggttctgctgacct  cggcgttacgtgcgtattcc |
| *talB* | talB-F  talB-R | tcccttcgtcagtacaccacc  aggcgacagcatcatcaatc |
| *ppsA* | ppsA-F  ppsA-R | tcagcaggaaaccttcctcaa  gataagagatggcgcgatcg |
| *aroA* | aroA-F  aroA-R | atggcaacagaactgcgtaa  catttgggatcaagaatcgtc |
| *aroB* | aroB-F  aroB-R | cgaaaccctggctcctctgt  cgtcgtcgggacttgaatga |
| *aroC* | aroC-F  aroC-R | ctttggcgaagaagttgagatga  ccgttgccgtaacaggtgat |
| *aroL* | aroL-F  aroL-R | gcccttgccgattcgcttaa  tcccgcccactcttcccttt |
| *aroK* | aroK-F  aroK-R | gagttgaccgagaaacaggg  ctcttcatacagcggattgc |
| *aroE* | aroE-F  aroE-R | ggtaaaggtgcgaatgtgac  cgtaaaccagggcggataaa |
| *ydiB* | ydiB-F  ydiB-R | tctttaaccgtcgggatgag  gcctgctgcaataacttcgt |
| *pheA* | PheA-F  PheA-R | catctggacgcccattacat  caatcggtacgacggcatag |
| *tyrB* | tyrB-F  tyrB-R | ggaatcaggcgtctgggtca  gccgttagtcgcttcgtcata |

**Legends to figures**

Figure s1

Fermentation consequence of the *tyrR* knocked out strain. The gray column means OD_600_ and the black column means L-Phe titer.

Figure s2

Sequence alignment results of the TyrR_wt_ and TyrR_mut_. This result was obtained by DNAMAN software.

Figure s3

Overexpression of the precursors’ synthesis encoded genes. The gray column means OD_600_ and the black column means L-Phe titer.

Figure s4

Relative strength of five promoters that were used to overexpress AroD in xllp08.

Figure s1





Figure s2


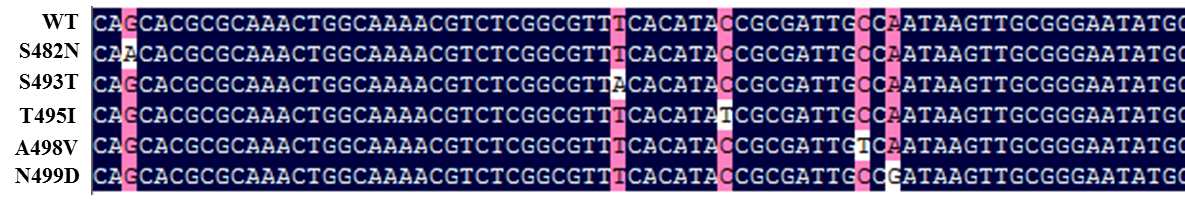


Figure s3





Figure s4


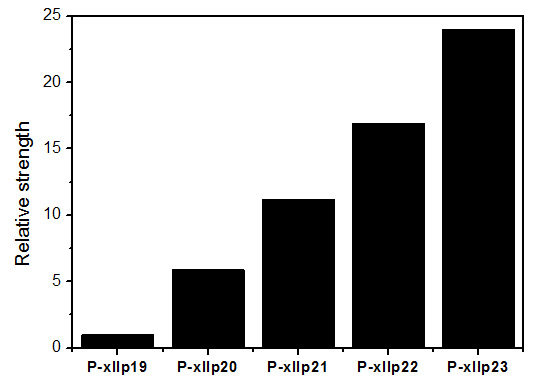

Supplement: Supplementary file 1 — Primers used in the genetic engineering. Table S2. The gradient variation ratio of phases A:B in HPLC analysis of L-Phe concentration. Table S3. Primers used in the Real-time PCR analysis. Figure S1. Fermentation consequence of the tyrR knocked out strain. The gray column means OD600 and the black column means L-Phe titer. Figure S2. Sequence alignment results of the TyrRwt and TyrRmut. This result was obtained by DNAMAN software. Figure S3. Overexpression of the precursors’ synthesis encoded genes. The gray column means OD600 and the black column means L-Phe titer. Figure S4. Relative strength of five promoters that were used to overexpress AroD in xllp08. (DOCX 582 kb) [file 12896_2018_418_MOESM1_ESM.docx]
